# Approaching the Humerus, Elbow, and Proximal Forearm Using the Trimano Arm Holder

**DOI:** 10.7759/cureus.58833

**Published:** 2024-04-23

**Authors:** Angelos Assiotis, Adam Rumian, Clarence Yeoh, Harpal S Uppal

**Affiliations:** 1 Trauma and Orthopaedics, Lister Hospital, East and North Hertfordshire NHS Trust, Stevenage, GBR

**Keywords:** supine position, elbow infection, olecranon fractures, midshaft humerus fractures, upper extremity trauma, elbow trauma, upper limb surgery

## Abstract

Upper limb surgeons frequently encounter complex cases involving the proximal humerus, elbow joint, and proximal forearm, both in trauma and elective practice. Given the diverse pathology in these areas, various surgical approaches have been described, each with its advantages, limitations, and specific patient positioning requirements.

We describe an operative technique that modifies the use of an existing, commercially available, dynamic pneumatic limb positioner, the TRIMANO FORTIS^®^ (Arthrex, Maquet GmbH), for open and arthroscopic procedures of the elbow, proximal forearm, midshaft, and distal humerus. This technique offers simplicity, reproducibility, and enhanced surgical efficiency.

## Introduction

Limb positioners have been in use for some time, both in upper and lower limb surgery [1,2]. They are useful in maintaining an appropriate position of the limb, with or without traction, while freeing up the surgical assistant, who would otherwise be manipulating the limb, to participate in other tasks during the procedure. Such positioners may be categorized as static or dynamic. Static devices usually present as L-shaped bars, arm-boards (also known as arm-tables), or gutter arm-holders, all of which are attached to the operating table via clamps.

The elbow joint is one of the most intricate joints in the human body, featuring three distinct articulations: the ulno-trochlear joint, the radio-capitellar joint, and the proximal radio-ulnar joint. Given the myriad pathologies associated with this complex joint, it is unsurprising that a multitude of surgical approaches have been devised to address them. Patterson et al. described 15 distinct approaches to the elbow in 2000 [3], and since then, additional approaches have emerged and gained traction, such as the para-olecranon approach [4] and the modified Boyd approach [5]. Predominantly, these approaches are posterior, global, lateral, and or/medial, with only a few indications for anterior approaches to the elbow joint.

While access to the anterior elbow typically involves positioning a patient supine with an arm-board, access to the rest of the elbow may be achieved with a patient in supine, lateral, or prone positions. A significant consideration that is taken into account when working on the humerus and elbow is that positioning significantly impacts the ease of fracture reduction and ligament repair in cases of elbow instability. For instance, repairing the lateral ulnar collateral ligament with a patient in a supine position on an arm-board necessitates an assistant maintaining elbow reduction. However, this often results in the elbow being subjected to valgus strain unless the shoulder is flexed and the hand is positioned near the patient’s mouth. Maintaining such a complex position heavily relies on the skills and patience of the surgical assistant.

Our proposed method offers reproducibility and safety and eliminates the need for an assistant dedicated solely to maintaining arm position. Moreover, it enables regular limb repositioning to facilitate posterior, medial, and lateral access during procedures.

## Technical report

**Figure 1:**
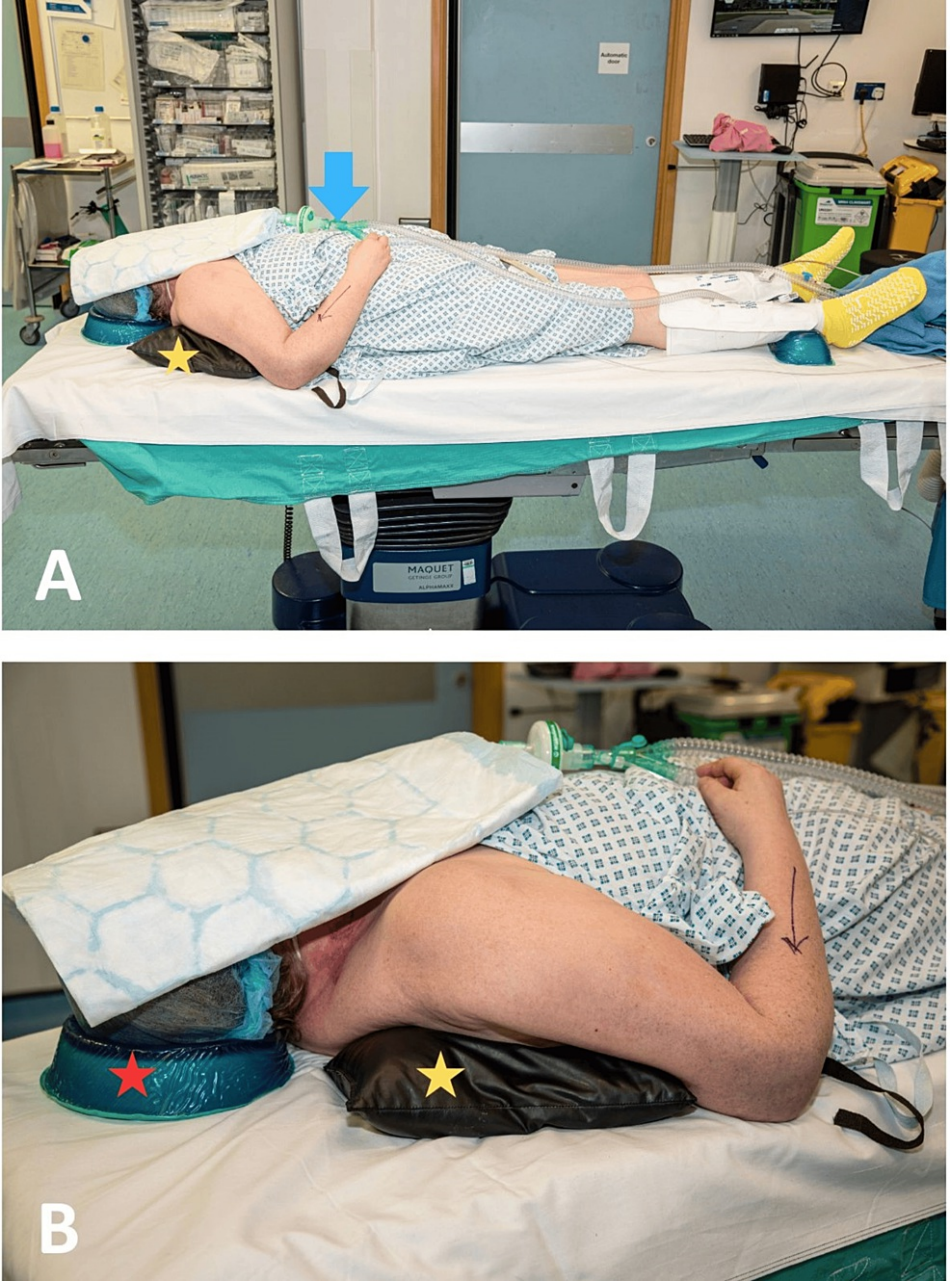
Patient placement on the operating table. A: The patient is supine on the operating table. There is a long-circuit breathing system (blue arrow) in place. Note the small sandbag under the injured upper limb (yellow star). B: Close-up image of the patient on the operating table, demonstrating the appropriate position for the sandbag (yellow star) and the head ring (red star).

Patients are positioned supine on the operating table, with a long-circuit breathing system connected to the anaesthetic machine. In cases involving smaller patients, a slight shift away from the operative side towards the fixed arm holder is recommended. A small sandbag is placed under the ipsilateral shoulder on the operative side to elevate the limb. Additionally, a head ring is positioned under the patient’s head, and the operating table is adjusted so that the patient’s head is opposite to the anaesthesia machine, allowing for proper placement of the image intensifier and preventing equipment contamination (Figure 1).

**Figure 2:**
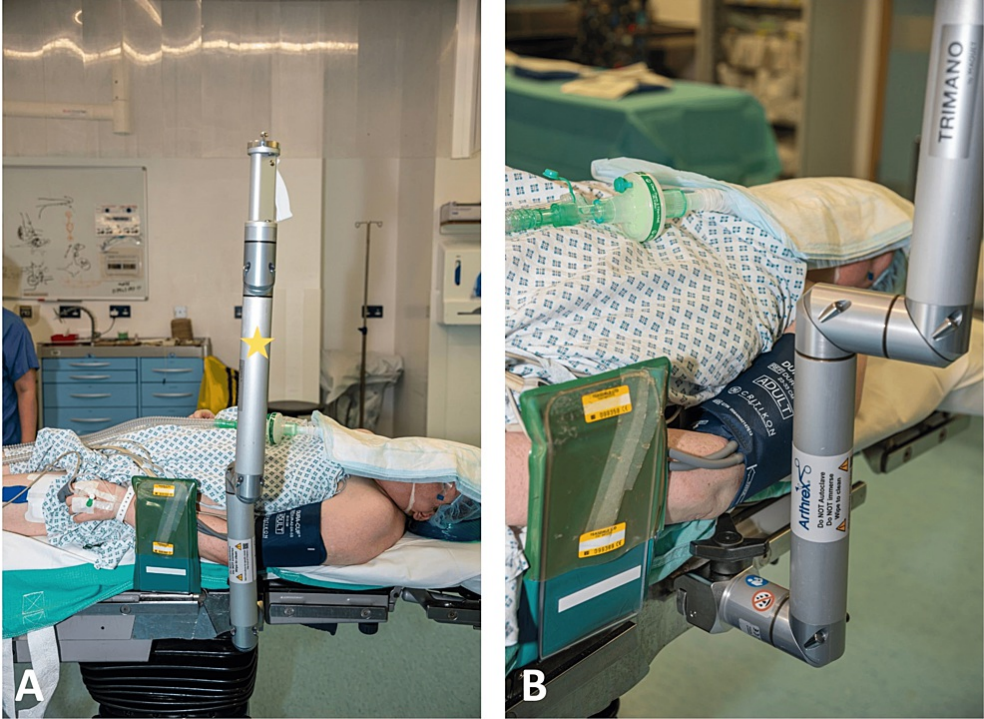
Placement of the TRIMANO arm holder on the operating table. A: The TRIMANO arm holder (yellow star) is placed on the opposite side of the injured limb, just distal to the level of the axilla. B: Close-up image of the TRIMANO arm holder, demonstrating its attachment to the operating table via its clamp, distal to the level of the patient’s axilla.

The TRIMANO FORTIS® (Arthrex, Maquet GmbH, hereafter described as the ‘TRIMANO’) arm holder is placed on the opposite side of the injured upper limb, just distal to the patient’s axilla (Figure 2).

**Figure 3:**
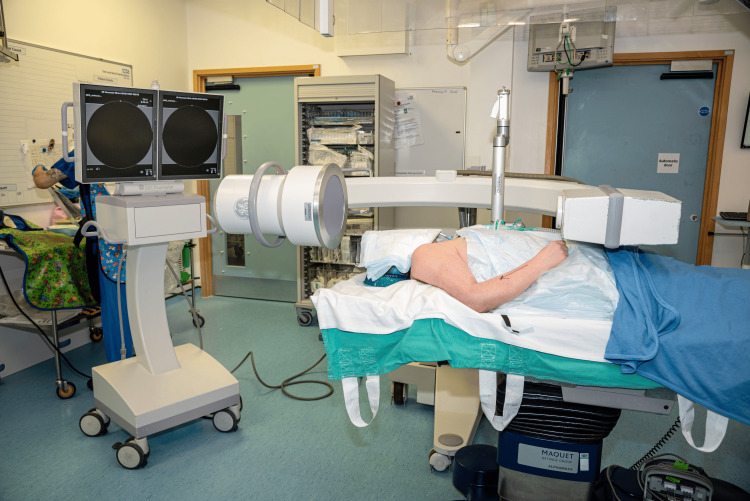
Positioning of the image intensifier and monitor. The image intensifier is placed in a horizontal position, with the detector proximal to the patient’s head. The intensifier should be draped with the appropriate sterile covers, as it will remain in the surgical field for the duration of the procedure. The monitor is placed proximal to the patient’s head.

The surgeon should instruct the radiographer to align the image intensifier horizontally, with the detector placed proximal to the patient’s head (Figure 3).

This configuration facilitates vertical movement of the image intensifier, as required, minimizing the risk of impingement between the device and the patient’s abdomen. When requesting any vertical or horizontal movement of the image intensifier, the surgeon should remain vigilant of the relative position of the device to the patient’s body. We routinely place our hand on the patient’s abdomen so that we can ensure that the patient’s abdomen will not come in contact with the image intensifier when this moves.

**Figure 4:**
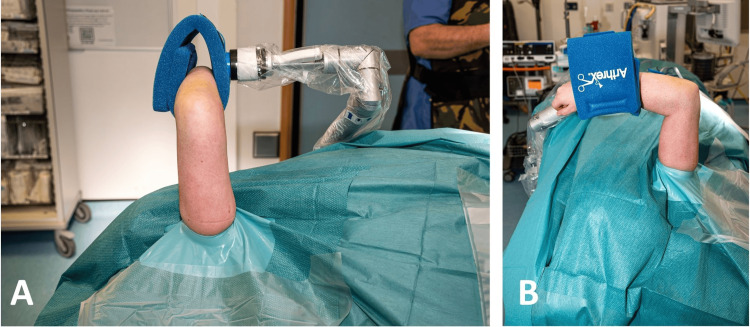
The injured limb is prepared and draped. A: Image demonstrating the view and access that the surgical team have of the patient’s injured limb after preparing the skin and draping. B: Image demonstrating the view of the injured limb from the anaesthetic machine. This shows the extensive access that this position allows for the humerus, elbow, and proximal forearm.

Following skin preparation, an extremity drape is applied, and a sterile tourniquet is utilized if necessary. The TRIMANO cover and disposable kit designed for elbow procedures (TRIMANO Elbow Kit Sterile Drape and Foam Arm Holder, AR-1646) are then attached to the pneumatic arm holder. The forearm is then placed in the disposable arm holder and secured. From this point in the procedure, there is no need for an assistant to support or move the arm (Figure 4).

**Figure 5:**
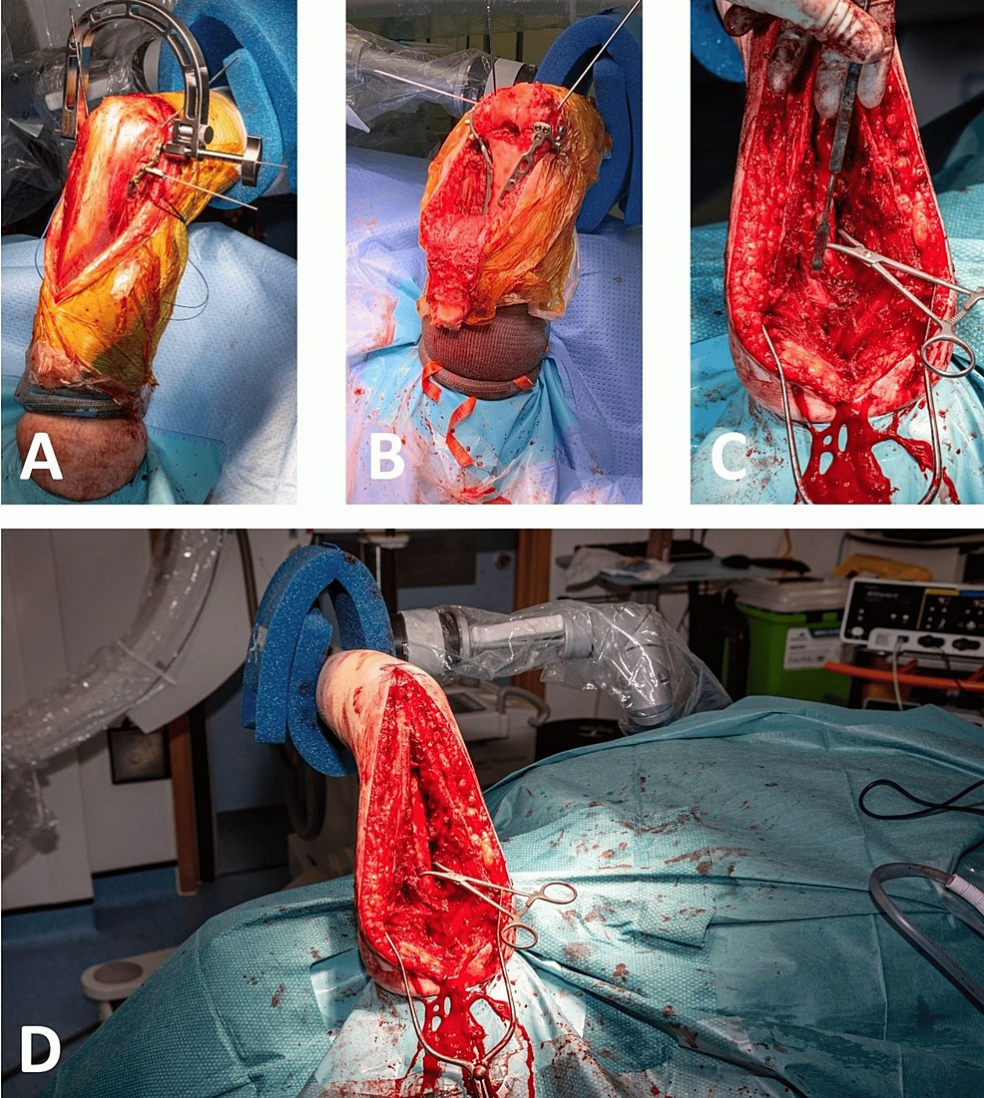
Images from posterior-based approaches to the humerus. A: Para-tricipital approach for the fixation of intra-articular distal humerus fracture with medial and lateral plates. B: Olecranon osteotomy approach for the fixation of highly comminuted fracture of the distal humerus with medial and posterolateral plates. C: Triceps-splitting approach for the fixation of humeral shaft fracture. The McDonald dissector is pointing to the radial nerve. D: Triceps-splitting approach for the fixation of distal humerus fracture, demonstrating the view obtained from our described patient positioning technique and the ease of reduction with a pair of fracture reduction forceps.

For posterior upper limb approaches and procedures, the humerus is positioned upright. It is employed for approaches such as para-tricipital, para-olecranon, olecranon osteotomies, and triceps splitting approaches (Figure 5).

**Figure 6:**
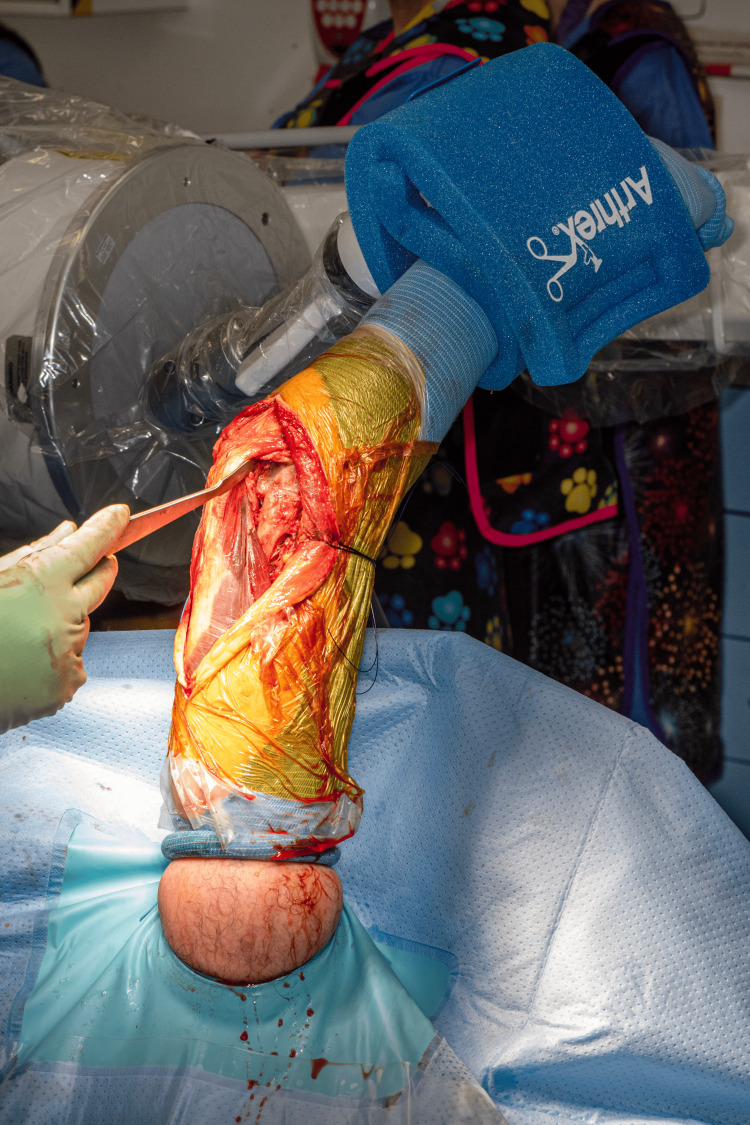
Image demonstrating elbow extension maintained by the TRIMANO arm holder. By extending the elbow, the triceps expansion can be retracted medially or laterally to achieve visualisation of the distal humerus. This is particularly helpful when stabilising fractures of the distal humerus through a ‘triceps-on’ approach.

By extending the elbow, the triceps muscle and tendon relax which facilitates better visualization of the humerus by allowing further retraction of the triceps (Figure 6).

**Figure 7:**
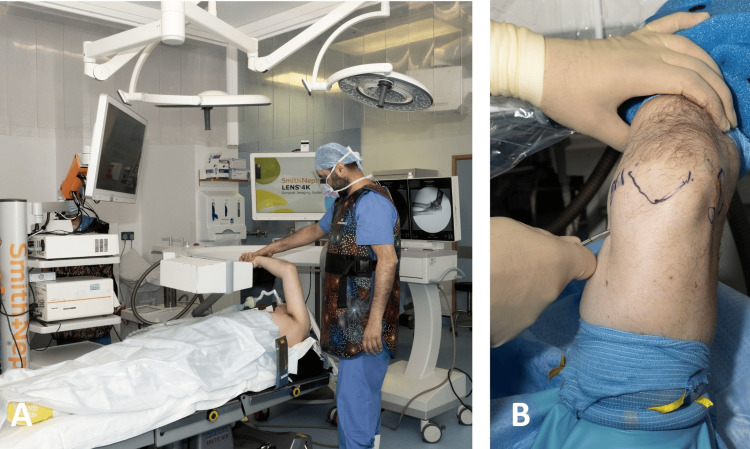
Elbow arthroscopic setup with our proposed technique. A: Image demonstrating the position of the patient, the two arthroscopic monitors, and the image intensifier. Please note that the arm is held in position by the surgeon, as it was not draped at that stage and not connected to the TRIMANO arm holder. B: Close-up image of the elbow attached in position by the TRIMANO arm holder while the surgeon creates the proximal anteromedial portal.

We use this position when performing elbow arthroplasty, partial or total, olecranon and proximal radial fracture fixations, distal and midshaft humeral fracture fixations, and, finally, elbow arthroscopy (Figure 7).

**Figure 8:**
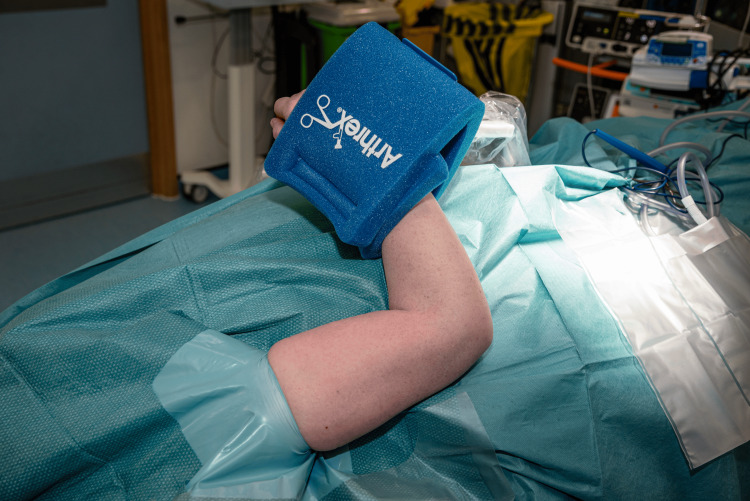
Image of prepared and draped upper limb in position for a lateral-based approach to the elbow. By placing the arm on the patient’s abdomen, the lateral aspect of the elbow is readily available to the surgical team.

For lateral elbow work, the arm holder and the patient’s arm are lowered to the patient’s abdomen (Figure 8).

This position allows for approaches and procedures to the radial head (surgical fixation or replacement) and the lateral humerus, including stabilisation work and lateral column procedures.

**Figure 9:**
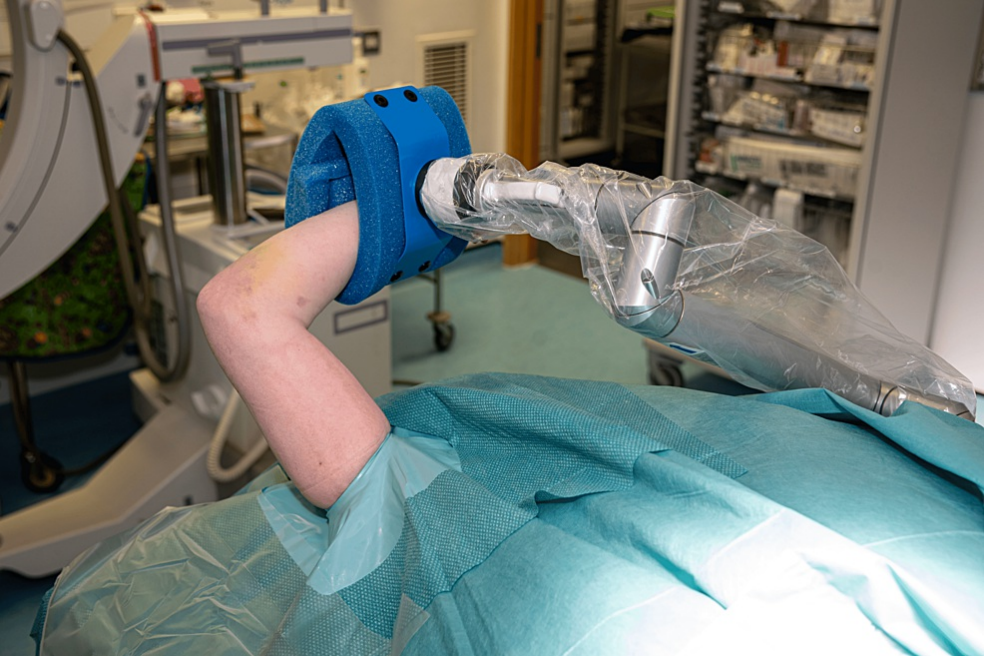
Image demonstrating the arm position for medial-based approaches to the elbow. By flexing the shoulder and placing the patient’s elbow above the patient’s head, the medial elbow is easily accessible to the surgical team.

The shoulder is forward flexed and the arm is brought over the patient’s head to facilitate work on the medial elbow (Figure 9).

**Figure 10:**
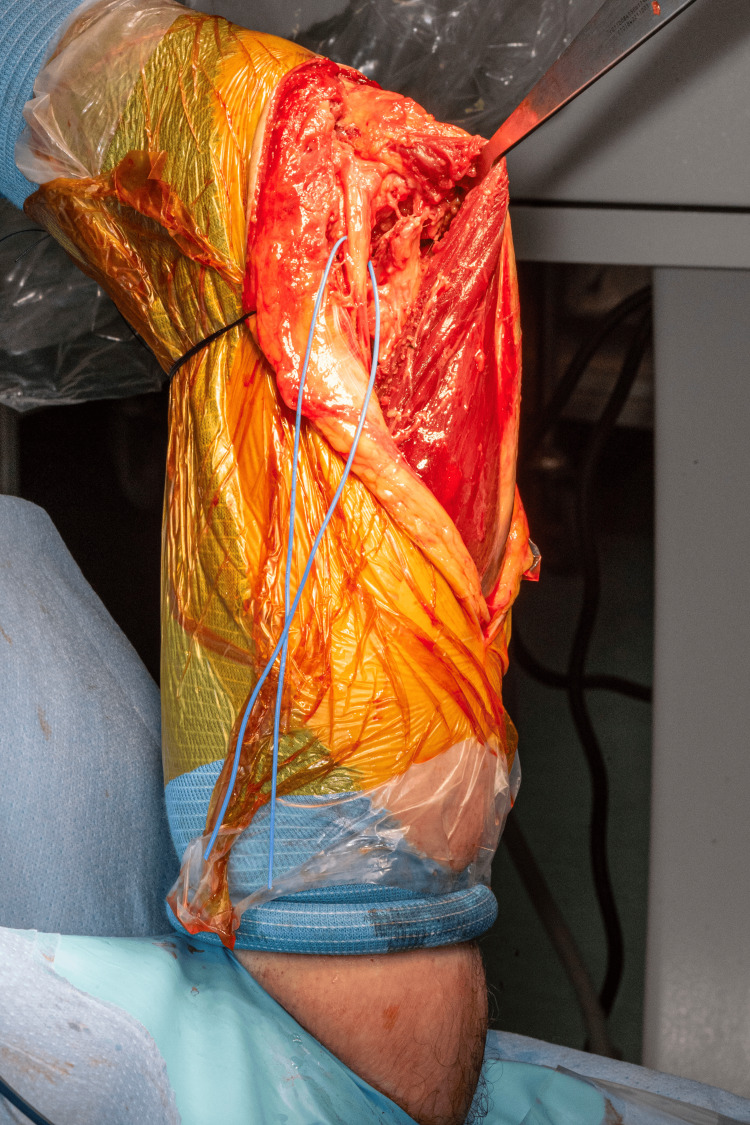
Image demonstrating the ulnar nerve from a para-tricipital approach to the distal humerus. The blue vascular sling is around the ulnar nerve, which was released at the level of the cubital tunnel.

This position allows for procedures involving the ulnar nerve, such as decompression and transposition and the medial elbow, such as procedures to the coronoid process and the medial collateral ligament (Figure 10).

**Figure 11:**
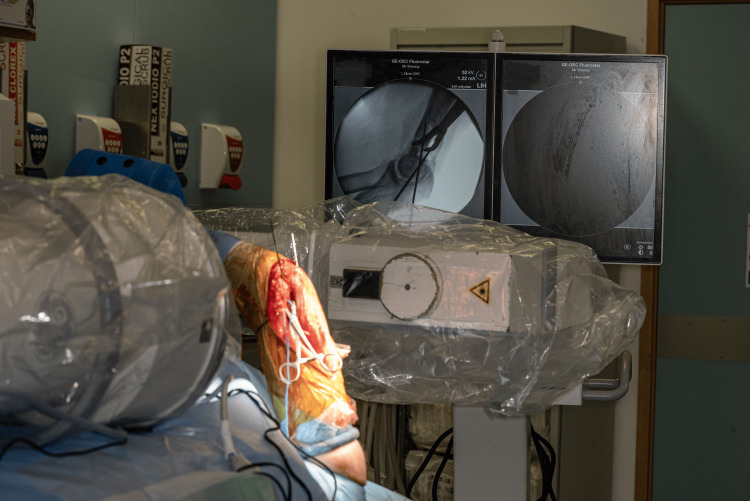
Image demonstrating a true lateral radiograph of the elbow. The proposed technique allows for true lateral views of the elbow without the need for the surgeon or an assistant to maintain the positioning of the arm.

Regarding intraoperative imaging, we can efficiently obtain high-quality lateral elbow radiographs ( Figure 11) in a tension-free manner with no varus or valgus strain, something that is not always easily achievable in patients with stiff shoulders in the supine position.

**Figure 12:**
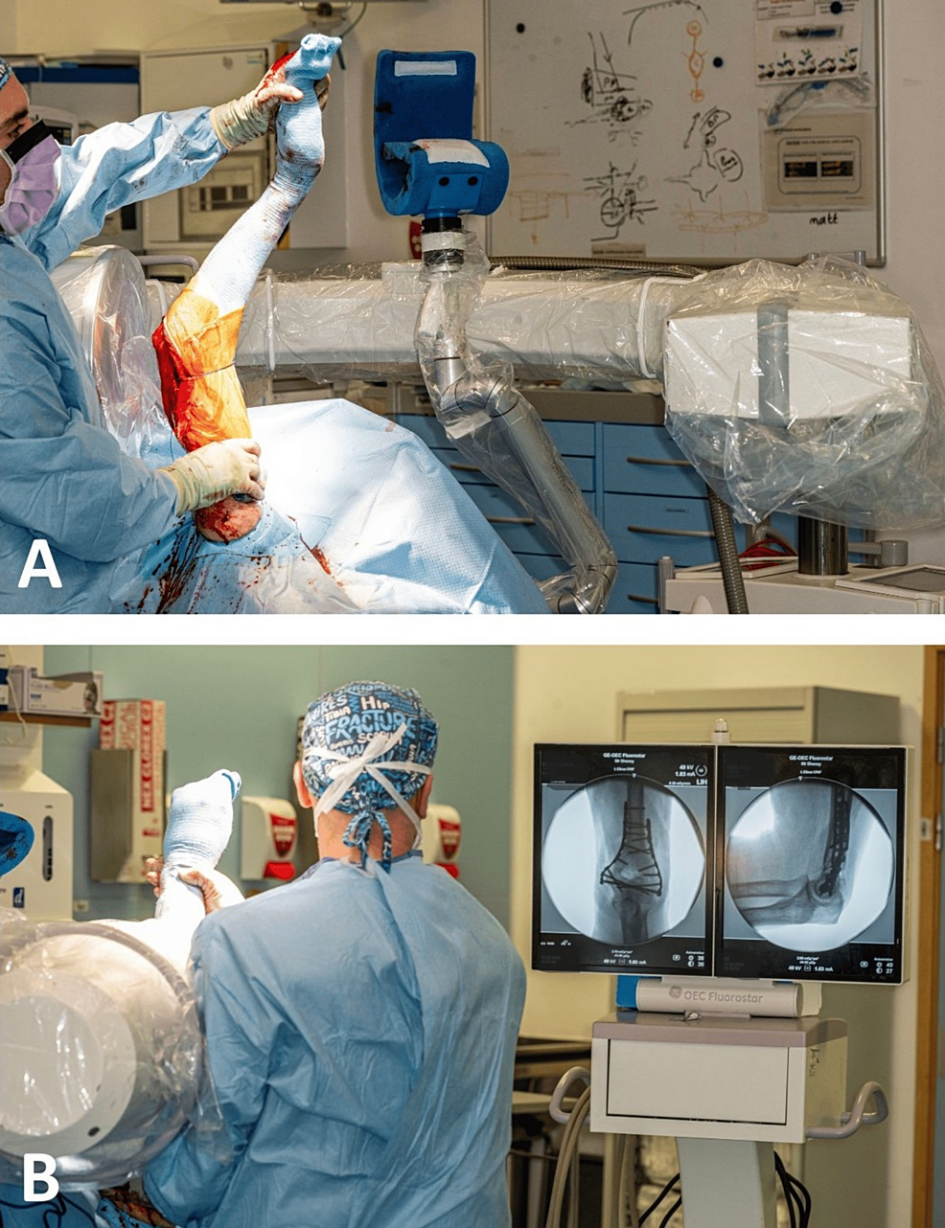
Image demonstrating an anteroposterior radiograph of the elbow. A: The surgeon removes the arm out of the arm holder and internally rotates the shoulder. B: This position allows for a high-quality anteroposterior radiograph of the elbow or distal humerus.

For the anteroposterior view, the arm is taken out from the TRIMANO arm holder by the surgeon and the shoulder is rotated so that the appropriate image is obtained (Figure 12).

In our unit’s experience with this technique, over 300 procedures have been performed without any position-related complications.

## Discussion

The TRIMANO device is routinely used as a limb positioner for procedures to the shoulder, elbow, and wrist, with the appropriate disposable attachments. The specific attachment that we use in our described technique was originally designed and introduced for procedures to the elbow by supporting the upper arm at the level of the midshaft humerus and allowing the forearm to suspend off it. This, however, does not allow for positioning the elbow in the various positions that we have already described and restricts elbow positioning similarly to static positioners such as L-shaped bars and gutters. Additionally, the manufacturer’s method prevents access to the humerus due to the attachment’s placement at the mid-humerus level. Hunter [6] previously described supine elbow arthroscopy using a static arm holder, but in that technique, the forearm is held with a static positioner which is applied on the operative side of the patient. Chen et al. described a different technique of supine elbow arthroscopy with an articulating arm holder placed on the operative side in proximity to the surgical team [7]. Camp et al. also proposed a technique for supine patient placement with the arm in a mechanical arm holder and the humerus in a perpendicular position in 2016 [8]. We believe that our suggested technique improves patient access as the limb positioner is not in close proximity to the surgeon and we are able to rapidly and efficiently change the position of the elbow with the use of the pneumatic arm holder. Moreover, our described positioning technique is identical for open and arthroscopic cases and allows for intraoperative image intensifier use, as described in the Technique section. A patient position for supine open elbow surgery was described by Wijeratna et al. in 2012 [9] using a static arm holder; this technique requires an assistant to move the limb and limits surgical exposure to the forearm because a gutter or an L-shaped bar is required.

Our suggested technique offers several other advantages. First, operating with the patient in the supine position allows for faster patient positioning compared to the prone position [10] and improved access to the airway [11], should that become necessary, compared to the prone or lateral decubitus position. It also allows for upper limb surgery to be safely performed in patients presenting with multiple injuries, whereas placing such patients in a lateral or prone position may be unsafe. Moreover, it eliminates the need for an assistant to support or move the arm during surgery. The vertical position of the humerus facilitates gravity-assisted blood drainage, enhancing visualization during elbow and humerus procedures. Another significant advantage is that when operating on the elbow for stabilisation surgery, the elbow is inherently placed in a stable position, as the weight of the forearm on a vertical humerus maintains the elbow reduced, while the ligaments are repaired or reconstructed. Similarly, when working on humeral shaft fractures, fractures are helpfully aligned by gravity and the reduction is more easily obtained and held during plate application. Finally, our technique eliminates the need for gutters or L-shaped bars, avoiding interference in radiographic images. Table 1 summarises the advantages of our described technique.

**Table 1:** Advantages of the described patient positioning technique.

Factor	Description
Airway access	Optimal
Positioning complications	Minimal
Positioning complexity	Easy
Demand for an assistant	No
Need for training	Yes (minimal)
Need for equipment	Yes
Intraoperative access	270-degree access to the elbow, proximal forearm, and distal/midshaft humerus
Static elbow positioning	In any position
Dynamic elbow positioning	Full flexion/extension and access to the posterior/medial/lateral elbow
Can be used with an arm-board	Yes
Can be used with a tourniquet	Yes
Can be used with an image intensifier	Yes
Allows conversion from arthroscopic to open surgery	Yes

However, there are some disadvantages to consider. Specialised equipment is required, potentially limiting its adoption in units lacking the TRIMANO arm holder. Of course, if a unit already has the TRIMANO arm holder on the shelf for use in shoulder surgery, they can easily expand its use for elbow/humerus/forearm cases, as we described above. Additionally, access to the anterior elbow is restricted while using the arm positioner, necessitating the use of an arm-board if a direct anterior approach is needed. While a 360-degree approach to the elbow and distal humerus is rarely needed, it is important to acknowledge that all positions pose restrictions in accessing various arm parts and obtaining appropriate images. Lastly, there is a short learning curve associated with working in this position and interpreting the images obtained on the image intensifier.

## Conclusions

To our knowledge, this technique has not been described before in the literature. It is reproducible and safe and allows for open and arthroscopic surgery to the elbow, humerus, and proximal forearm for posterior, lateral, and medial approaches. Having used this technique in more than 300 procedures in the upper limb, we believe that it presents significant improvements over previously described methods and we advocate for its widespread adoption, particularly in units equipped with the TRIMANO arm holder.
